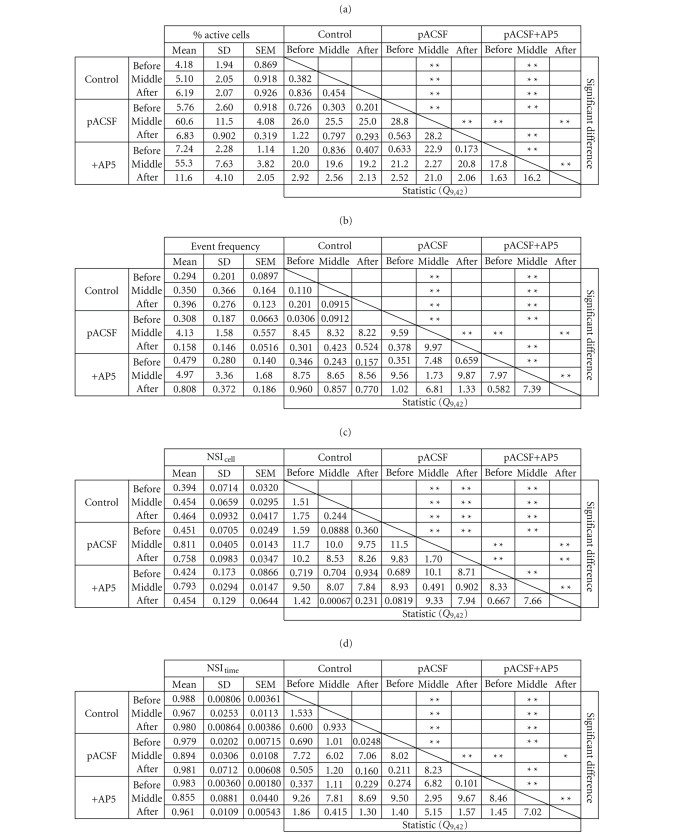# Spontaneous Plasticity of Multineuronal Activity Patterns in Activated Hippocampal Networks

**DOI:** 10.1155/2008/108969

**Published:** 2008-07-14

**Authors:** Atsushi Usami, Norio Matsuki, Yuji Ikegaya

**Affiliations:** ^1^Laboratory of Chemical Pharmacology, Graduate School of Pharmaceutical Sciences, The University of Tokyo, Tokyo 113-0033, Japan; ^2^Precursory Research for Embryonic Science and Technology (PRESTO), Japan Science and Technology Agency, 5 Sanbancho Chiyoda-ku, Tokyo 102-00075, Japan

## Abstract

Using functional multineuron imaging with single-cell resolution, we examined how hippocampal networks by themselves change the spatiotemporal patterns of spontaneous activity during the course of emitting spontaneous activity. When extracellular ionic concentrations were changed to those that mimicked in vivo conditions, spontaneous activity was increased in active cell number and activity frequency. When ionic compositions were restored to the control conditions, the activity level returned to baseline, but the weighted spatial dispersion of active cells, as assessed by entropy-based metrics, did not. Thus, the networks can modify themselves by altering the internal structure of their correlated activity, even though they as a whole maintained the same level of activity in space and time.

## 1. INTRODUCTION

The brain is an enormously complex system that is composed of diverse types of neurons interacting with one another through topologically defined networks. A fundamental feature of neuronal networks is plasticity, that is, its functional connectivity undergoes an activity-dependent modification, and this change persists over time. Experimentally, plasticity is usually induced by artificial repetitive stimulation, such as tetanic stimulation, low-frequency stimulation, or repeated coactivation of presynaptic and postsynaptic neurons [1–8]. In the intact brain, however, neuronal networks spontaneously modify themselves, depending on sensory-evoked or internally generated activity. Such intrinsically occurring plasticity is poorly understood, compared to artificially induced “conventional” synaptic plasticity.

In this study, we addressed how neuronal networks undergo plastic changes while they generate internal activity, by using functional multineuron calcium imaging (fMCI), an optical recording technique with calcium-sensitive fluorescent indicator to monitor action potentials from large neuron populations [9–18]. Unlike electrophysiologic techniques, including extracellular unit recordings, fMCI allows us to detect not only the activity of active neurons but also the silence of nonactive neurons (cf. single-unit or multiunit recordings cannot determine how many neurons are silent). Thus, fMCI can more comprehensively capture the pattern of multineuronal activity in a local network.

We sought to examine how the inner structure of spontaneous activity changes at the network level following transiently enhanced spontaneous activity. This idea has originated from our recent findings that briefly increased levels of spontaneous activity induce long-lasting plasticity of synaptic transmission in hippocampal CA3 region without any electrophysiological stimulation [19]. These findings suggest that active networks update their internal states in the course of generating spontaneous activity. In this previous study, however, artificial electric pulses were applied to afferent fibers as “test” stimulation to monitor the strength of synapses, and it remains to be addressed how internally generated activity itself is modified by spontaneous activity.

In the present study, therefore, we have designed a series of experiments without any artificial stimulation in order to examine the effect of enhanced levels of spontaneous activity on the spatiotemporal pattern of spontaneous activity. To this end, we have introduced a simple entropy-based metrics, that is, a modified measure of the so-called Shannon index (SI), which is widely used in the field of ecology for measuring the extent of diversity of species living in a region [20]. With this new metrics, spontaneously occurring network plasticity can be detected, although other conventional parameters, such as active cell numbers and spike rates, cannot capture the network plasticity.

## 2. MATERIALS AND METHODS

### 2.1. Materials

Oregon Green 488 BAPTA 1-AM and Pluronic F-127 were obtained from Invitrogen (Carlsbad, Calif, USA). Cremophor EL and d,l-2-amino-5-phosphonopentanoic acid (AP5) were obtained from Sigma (St. Louis, Mo, USA). The stock solutions were stored at −20°C and diluted immediately before use.

### 2.2. Slice culture preparations

Hippocampal slice cultures were prepared from postnatal day 7 Wistar/ST rats (SLC, Shizuoka, Japan) as previously described [21], according to The University of Tokyo guidelines for laboratory animal care and safety. Briefly, rat pups were chilled and decapitated. The brains were rapidly removed and cut into horizontal 300-*μ*m-thick slices using a DTK-1500 microslicer (Dosaka, Kyoto, Japan) in aerated, ice-cold Gey's balanced salt solution (Invitrogen, Gaithersburg, Md, USA) supplemented with 25 mM glucose. Entorhino-hippocampal stumps were cultivated on Omnipore membrane filters (JHWP02500, Millipore, Bedford, Mass, USA) that were laid on O-ring plastic disks (Hazai-Ya, Katsushika-ku, Tokyo). Cultures were fed with 1 mL of 50% minimal essential medium, 25% Hanks' balanced salt solution (Invitrogen), 25% horse serum (Cell Culture Laboratory, Cleveland, Ohio, USA), and antibiotics in a humidified incubator at 37°C in 5% CO_2_. The medium was changed every 3.5 days.

### 2.3. fMCI

fMCI was performed as previously described [18]. Briefly, slices at day 8-to-12 in vitro were washed three times with artificial cerebrospinal fluid (ACSF), bubbled with 95%**O_2_ and 5% CO_2_, consisting of (mM): 127 NaCl, 26 NaHCO_3_, 1.5 KCl, 1.3 KH_2_PO_4_, 1.4 MgSO_4_, 2.4 CaCl_2_, and 10 glucose. They were transferred into a 35-mm dish filled with 2 mL of dye solution and incubated for 60 minutes in a humidified incubator at 37°C in 5% CO_2_. The dye solution was ACSF containing 10 *μ*l of 0.1% OGB-1 AM/DMSO, 2 *μ*l of 10% Pluronic F-127/DMSO, and 2 *μ*l of 5% Cremophor EL/DMSO. After being washed, slices were incubated at room temperature at least for 30 minutes in fresh ACSF. They were transferred to a recording chamber perfused with 32°C ACSF at a rate of 1.5–2.0 mL/min. After 10 minutes, the activity was imaged from the hippocampal CA3 region for 5 minutes. Then the extracellular solution was replaced with physiologic ACSF (pACSF) consisting of (mM): 127 NaCl, 26 NaHCO_3_, 3.3 KCl, 1.24 KH_2_PO_4_, 1.0 MgSO_4_, 1.0 CaCl_2_, and 10 glucose [17, 19, 22, 23]. After 15 minutes, the extracellular solution was replaced with normal ACSF. As a result, calcium transients evoked by spontaneous spike activity were constantly recorded for 30 minutes in total. Images (16-bit intensity, 512 × 512 pixels, 742 × 742 *μ*m^2^) were captured at 10 frames/s with a Nipkow-disk confocal unit (CSU22, Yokogawa Electric, Tokyo, Japan), cooled CCD camera (iXon DV887DCS-BV; Andor Technology, Belfast, UK), upright microscope (Eclipse FN1; Nikon, Tokyo, Japan), water-immersion objective (16×,0.80 numerical aperture, CFI75LWD16XW; Nikon, Tokyo, Japan), and image acquisition software (SOLIS; Andor Technology, Belfast, UK). Fluorophores were excited at 488 nm with an argon-krypton laser (10–15 mW, 641-YB-A01; Melles Griot, Carlsbad, Calif, USA) and visualized through a 507 nm long-pass emission filter. Spike-triggered calcium signals were semiautomatically detected with custom-written software in Visual Basic Version 6.0 (Microsoft, Seattle, Wash, USA) [12] and inspected by eye.

### 2.4. Electrophysiological recordings

Loose-patch-clamp recordings were performed with glass pipettes filled with pACSF to record extracellular single-unit activity. Recordings were carried out with Axopatch 700B amplifiers (Molecular Devices, Union City, Calif, USA), and signals were low-pass filtered at 1 kHz, digitized at 10 kHz, and analyzed with pCLAMP version 10.0 (Molecular Devices).

### 2.5. Data analysis of multineuronal activity

SI quantifies the dispersion of components in a histogram [20]. SI is defined as

(1) $$\text{SI}\;=\;-\Sigma_{i}(\frac{k_{i}}{K})\log_{2}(\frac{k_{i}}{K}),$$

where *K* is the total number of components, and *k*_*i*_ is the number of components in the *i*th bin. This definition of diversity is conceptually equivalent to Shannon's entropy. Because SI is very sensitive to *K* and the bin size, SI has often been normalized with the maximal value and other standard values to compare groups [24, 25]. Here we normalized SI with the maximal (SI_max_) and minimal values (SI_min_) that can be taken. SI_max_ and SI_min_ were obtained through data shuffle with maintaining *K* and bin. SI_max_ is given when components are as evenly redistributed as possible, whereas SI_min_ is given when components are as biased as possible (Figure 2(a)). Then normalized SI (NSI) is defined as

(2) $$\text{NSI}\;=\;\frac{\text{SI}\;-\;{\text{SI}}_{\min}\,}{{\text{SI}}_{\max}\,\;-\;{\text{SI}}_{\min}}.$$

Thus, it takes a value from 0 to 1, with higher values being more dispersive. Unless otherwise specified, the NSI value was used to quantify the dispersion of activity in a multineuronal spike train. The dispersion was evaluated in two scopes, that is, the vertical (spatial) and horizontal (temporal) projections in a rastergram, which were referred to herein as NSI_cell_ and NSI_time_, respectively. A 1-minute window was placed at a given time and shifted at a step of 30 seconds to scan the temporal dynamics of the NSIs (Figure 2(c)).

## 3. RESULTS

**Figure 1:**
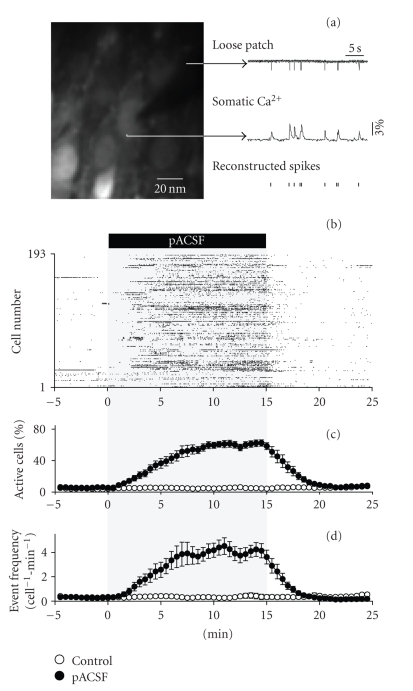
*Physiologically relevant ionic conditions induce a reversible increase in spontaneous activity of hippocampal CA3 neurons in organotypic cultures*. (a) Simultaneous loose patch-clamp recording and time-lapse imaging of somatic calcium signal from a hippocampal CA3 neuron loaded with Oregon green 488 BAPTA-1AM. The timings of action potentials can be reconstructed from the onset timings of individual calcium rise events. (b) Representative example of a change in CA3 network activity following 15-min replacement of normal extracellular solution with pACSF. Each dot represents a single-calcium transient. (c) pACSF-induced change in the percentage of active cells to the total number of cells monitored. (d) pACSF-induced change in the mean event frequency per cell. Open and closed circles indicate group without (*N* = 5 slices, Control) and with pACSF replacement (*N* = 8 slices, pACSF), respectively. Data are means ± SEMs.

Hippocampal slice cultures were incubated with Oregon green 488 BAPTA-1AM, and the fluorescent intensity from the cell bodies of CA3 neurons was measured at 10 frames per second with spinning-disk confocal microscopy. Because action potentials, as assessed by loose-patch-clamp recordings, were reflected in transient rises in somatic fluorescent signal (Figure 1(a)), we were able to reliably reconstruct the time series of action potentials from calcium fluorescent traces. Spontaneous activity was recorded from, on average, 157 ± 57 neurons (mean ± SD of 17 slices), ranging from 101 to 350 neurons.

In conventionally used “control” ACSF, only about 5% neurons were spontaneously active (Figure 1(b)). The ionic composition of this standard ACSF is different from that of in vivo circumstances, because it was designed to reduce the level of spontaneous neuronal activity in slice preparations [26–33]. Indeed, when slices are perfused with solution with physiologically relevant ionic conditions (pACSF), the activity level is known to increase [17, 19, 22, 23]. In our preparation as well, more neurons became spontaneously active in pACSF (Figure 1(b)). After 15 minutes, pACSF was replaced again with control ACSF, and the activity level was returned to the pre-pACSF baseline within 5 minutes.

Data of 8 slices are summarized in Figures 1(c) and 1(d). Perfusion with pACSF increased the number of active cells (c) and the mean activity rate (cell^−1^ ·min^−1^) (d). The increases in the activity level were statistically significant, whereas there was no significant difference between the pre-pACSF period (−5–0 minute) and post-pACSF period (20–25 minutes) (Tables 1 (a) and 1(b)). In control experiments without pACSF perfusion, the level of spontaneous activity was kept stable throughout 30 minutes of optical imaging (Figures 1(c) and 1(d), Tables 1(a) and 1(b), *N* = 5 slices). This indicates that photodamage and photobleaching are negligible in our fMCI experiments [17].

**Figure 2:**
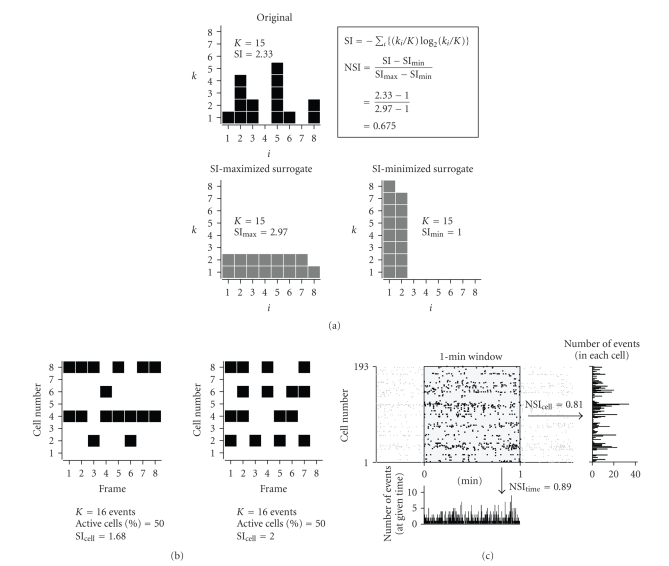
*Diagram of entropy-based metrics to capture network activity patterns*. (a) Shannon index (SI) is calculated with the Shannon entropy equation and normalized with its maximum and minimum values that can be taken through data shuffle with keeping the total number of events constant, as shown in the schematics. (b) SI can take different values depending on the pattern of activity, even when the numbers of activity events and active cells are invariant. (c) Example of the normalized SI (NSI) in multineuronal spike trains. A 1-minute window (shadow area) was placed at any given time on a rastergram. Two histograms were made by projecting the dataset to the vertical (right) and horizontal axes (bottom), so that NSIs evaluate the event dispersion in terms of space and time (NSI_cell_ and NSI_time_, resp.).

Was the effect of pACSF truly reversed? We sought to examine whether or not the history of the transiently enhanced activity is registered in some form of network activity. To this end, we introduced SI, new parameters to capture the diversity of the spatiotemporal pattern of network activity (Figure 2). Note that even though both the numbers of activity and active cells are unchanged, SI can take different values, reflecting the pattern of activity (Figure 2(b)). For data comparison, we normalized SI into NSI (see methods). We used two NSI, that is, NSI_cell_ and NSI_time_. NSI_cell_ reflects the spatial dispersion of calcium events across neurons, that is, NSI_cell_ becomes smaller when more events occur in a few specific neurons. On the other hand, NSI_time_ reflects the degree in temporal decorrelation of calcium events, that is, NSI_time_ becomes smaller when more events occur in synchrony across neurons (Figure 2(c)).

**Figure 3:**
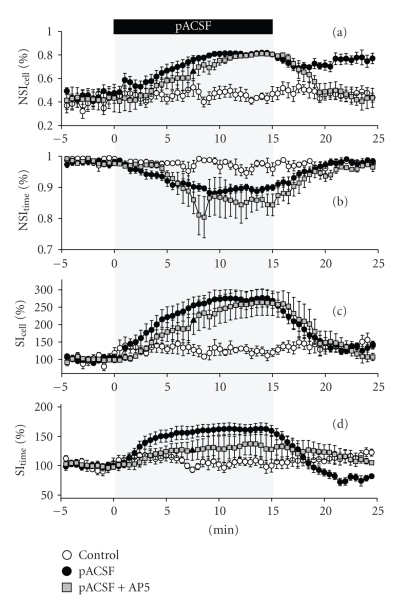
*pACSF induces an NMDA-dependent persistent increase in NSI_cell_, but not NSI_time_.* The same data as Figures 1(c) and 1(d) and data of perfusion with pACSF in the presence of 50 *μ*M AP5 were analyzed with the NSI_cell_ (a), NSI_time_(b), %SI_cell_ (c), and %SI_time_(d) indices (Control, *N* = 5; pACSF, *N* = 8; pACSF+AP5, *N* = 4). (a) and (b) pACSF-induced increase in NSI_cell_ persisted after replacement with normal ACSF, an effect that was prevented by AP5, whereas pACSF-induced increase in NSI_time_ was recovered to the pre-pACSF baseline level after pACSF washout. (c) and (d) show that neither %SI_cell_ nor %SI_time_ detected pACSF-induced plastic changes. Data are means ± SEMs.

pACSF perfusion led to an increase in NSI_cell_ (Figure 3(a)). This indicates that neurons in the network participated more uniformly in spontaneous activity. On the other hand, pACSF caused a decrease in NSI_time_ (Figure 3(b)), that is, neurons became to exhibit more synchronized activity. In general, NSI_cell_ and NSI_time_ may display a tradeoff change, that is, the coincident NSI_cell_ increase and NSI_time_ decrease as observed here seem to be plausible because network synchronization inevitably recruits a more number of neurons at a given time. In other words, pACSF-enhanced network activity was accompanied by an increase in synchronicity. Surprisingly, however, the NSI_cell_ increase was maintained even after pACSF washout, whereas the NSI_time_ decrease reverted to the pre-pACSF baseline within 5 minutes. This is intriguing, at least in two points, (i) a dissociation between NSI_cell_ and NSI_time_ parameters, and (ii) plasticity in the form of network activity patterns without a change in the activity level. Incidentally, these phenomena were not detected with another normalization of SI, in which SI was divided simply by the mean SI in the pre-pACSF period (Figures 3(c) and 3(d)).

This plasticity was dependent on *N*-methyl-D-aspartate (NMDA) receptor activity, because pACSF-increased NSI_cell_ did not persist in the presence of 50 *μ*M AP5, an NMDA receptor antagonist (Figures 3(a) and 3(b), *N* = 4 slices). In control experiments without pACSF perfusion, NSI_cell_ and NSI_time_ were unchanged for 30 minutes (Figures 3(a) and 3(b), *N* = 5 slices).

We summarize all statistically analyzed data in Table 1. Three groups were compared: control group (*N* = 5 slices), pACSF replacement groups without (*N* = 8 slices) and with AP5 (*N* = 4 slices). Four parameters, that is, the percentage of active cells to the total cells monitored (Table 1(a)), the mean event frequency per minute per cell (Table 1(b)), NSI_cell_ (Table 1(c)), and NSI_time_ (Table 1(d)), were extracted from three periods, that is, the pre-pACSF period (−5 to 0 minute relative to the start time of 15-minute perfusion with pACSF), the middle-pACSF period (10 to 15 minutes), and the post-pACSF period (20 to 25 minutes). The data were assessed with one-way analysis of variance (ANOVA) and post-hoc Tukey-Kramer multiple comparison test.

## 4. DISCUSSION

In a previous study using whole-cell patch-clamp recordings, we demonstrated that CA3 pyramidal neurons respond to pACSF perfusion by emitting spontaneous oscillatory activity and displaying bidirectional long-lasting synaptic modification [19]. The direction and amount of the plasticity varied depending on the patterns of spontaneous activity shown by the neuron and the locations of synapses, and thus pACSF-induced plasticity was diverse across neurons and experiments. In the previous study, however, synaptic responses were monitored with artificial electric stimulation, that is, bulk activation of presynaptic axon fibers, and spontaneous activity and plasticity were recorded from at most two neurons at once. Thus, it remained unclear how pACSF-induced enhancement of spontaneous activity affected the pattern of intrinsic network activity. In the present study, we described that pACSF-enhanced activity induced a change in the pattern of spontaneous activity toward an increase in NSI_cell_. In these experiments, we did not use any electric stimulation at all; both activity and plasticity were spontaneously generated by the CA3 network, and the active networks reported the occurrence of plasticity through their own activity.

**Figure 4:**
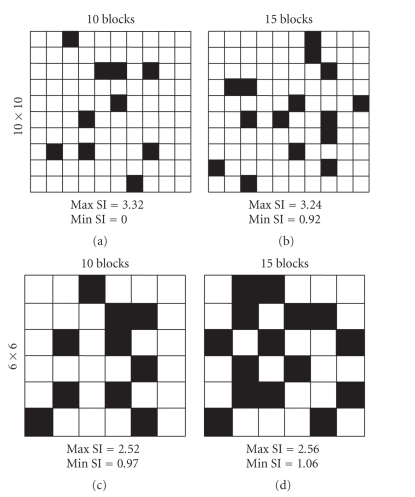
*The range of Shannon Index (SI) changes depending on the number of data points and the bin size*. As examples, when 10 (left) and 15 (right) blocks are distributed in the 10 × 10 (top) and 6 × 6 (bottom) squared spaces, the maximal and minimal SI values are shown in each case.

We underline that without NSI, it was difficult to detect plasticity in spontaneous activity. SI is an information theory-based metrics that has been widely used to assess the number and relative abundance of animal and plant species in ecology [20]. This measure was recently introduced to quantify the heterogeneity of GABAergic synaptic and cellular populations [34, 35]. A change in SI in peak conductance in injected inhibitory postsynaptic currents is associated with the firing rates of CA1 pyramidal neurons. Furthermore, an increase in SI of interneuron populations is linked to a decrease in network coherence, even when population variance remained unchanged. Thus, SI is a useful scalar to assess diversity in various experimental data. In our experimental systems, however, SI is susceptible to the total number of cells, the frequency of calcium transients, movie length, and bin size. For example, cases in which 10 or 15 data points are distributed in the 10×10 and 6×6 squares are shown in Figure 4. Note that the maximal and minimal SI values are different among these cases. Thus, SI cannot be directly compared between different datasets, especially with different numbers of neurons monitored or different levels of network activity. To overcome this problem, we normalized SI so as to be independent of these factors. In addition, as NSI ranges from 0 to 1, it is mathematically tractable. Using NSI, we succeeded in quantifying the pattern of network activity and thereby detecting plasticity hidden at the network level.

There are mainly two types of synaptic plasticity, NMDA receptor-dependent and NMDA receptor-independent plasticities [36]. Plasticity in NSI_cell_ was the former, because it did not occur in the presence of the NMDA receptor antagonist AP5. NMDA receptor-independent plasticity, such as L-type calcium channel-dependent, calcium-permeable AMPA receptor-dependent, and metabotropic glutamate receptor-dependent plasticity observed in pyramidal cells and interneurons [37–42], may occur during pACSF perfusion. Given that the post-pACSF activity level in the presence of AP5 was higher than the pre-pACSF level, NMDA receptor-independent plasticity may counteract NMDA receptor-dependent plasticity and balance the degree of spontaneous activity after pACSF washout.

## 5. CONCLUSIONS

Plasticity in the brain is essential in processing and storing information so that animals can act against changing environments in a predictive manner. The major form of neuronal plasticity is embodied through activity-dependent modification of synaptic connectivity or strength. Various forms of synaptic plasticity have been described at glutamatergic and GABAergic synapses in terms of direction, magnitude, duration, receptor and molecular mechanisms, and triggering stimuli. Many studies on synaptic plasticity, however, have focused on the behavior of monosynaptic transmission or single neurons by ignoring their complex dynamics in a network, or otherwise on the averaged response of a local network to stimulation by ignoring the detailed dynamics of individual neurons. Moreover, in these studies, extremely artificial stimuli, such as electrical or chemical stimulation, have been used to induce synaptic plasticity. In this scope, our experiments are designed to utilize fMCI to monitor network activity with single-cell resolution, use spontaneous activity as plasticity-triggering stimulation, and monitor the pattern of spontaneous activity. By introducing NSI, a new parameter to quantify event diversity, we found that a network experiencing transiently enhanced spontaneous activity modifies the inner structure of its spontaneous activity. Transiently enhanced activity caused a decorrelation of preexisting spontaneous activity, which might serve as a sparse memory trace decodable in downstream neuronal systems. Importantly, even though such dramatic plasticity occurred in the entire network, the observed level of spontaneous activity was preserved over time. We interpret this persistent increase in NSI as a reconfiguration of network dynamics, where a trace of the elevated activity period has remained, despite the alleged decrease in network activity. Therefore, this novel form of plasticity at the network level is featured by “homeostasis-like” scaling properties and may help avoid network hyperexcitability and hypoexcitability.

## Figures and Tables

**Table 1:** *Statistics of pACSF-induced plasticity of spontaneous network activity*. The percentage of active cells (a), the mean event frequency (b), NSI_cell_ (c), and NSI_time_ (d) are shown as the average values from −5 to 0 minute (before), from 10 to 15 minutes (middle), and from 20 to 25 minutes (after) after treatment of pACSF. (Control, *N* = 5; pACSF, *N* = 8; pACSF+AP5, *N* = 4).*P < .05, **P < .01 post-hoc Tukey-Kramer multiple comparison test after one-way ANOVA.